# Caveats for Using Shock Tube in Blast-Induced Traumatic Brain Injury Research

**DOI:** 10.3389/fneur.2013.00117

**Published:** 2013-08-26

**Authors:** Yun Chen, Shlomi Constantini

**Affiliations:** ^1^BrightstarTech, Inc., Clarksburg, MD, USA; ^2^Department of Pediatric Neurosurgery, Dana Children's Hospital, Tel Aviv Medical Center, Tel Aviv University, Tel Aviv, Israel

**Keywords:** blast, traumatic brain injury, shock tube, shock wave, reflected wave, Mach stem, rarefaction wave

Blast-induced traumatic brain injury (TBI) is currently an important and very “hot” research topic because it has been acknowledged to be a significant source of morbidity and disability during the wars in Iraq and Afghanistan, among blast victims. A total of 545 academic articles about blast TBI research have been published since 1946, of which 82% (447 articles) have been published since 2003, and 57% (312 articles) were published from 2010 to 2013. A number of experimental models are currently implemented to investigate the mechanisms of blast-induced TBI in rodents and larger animals such as rabbits and swine. As the fundamental shock wave generator, shock tubes (either compressed air-driven or detonation-driven) are generally employed in these experimental models.

The compressed air-driven shock tube is a horizontally mounted, circular steel tube, in which a gas at low pressure (the driven gas) and a gas at high pressure (the driver gas) are separated using diaphragms (such as polyester Mylar membrane). After the diaphragm suddenly ruptures at predetermined pressure thresholds (e.g., 126–147 kPa), shock waves are generated and propagate through the low pressure section (the driven section) toward the mouth of the shock tube. The detonation-driven shock tube is a cylindrical metal tube that is closed at one end. The blast, causing the shock waves, is generated by detonation of an explosive charge in the closed end of the tube.

Both compressed air-driven and detonation-driven shock tubes can produce blast shock waves to induce blast injuries in animals. However, because of their designs and structures, both shock tubes are not able to generate the Friedlander wave (an ideal form of a primary blast wave) that occurs when a powerful explosive detonates in a free field, without nearby surfaces that can interact with the wave. A series of complex shock waves are then generated following the lead shock wave (the original shock front), including reflected shock waves, a Mach stem, an unsteady turbulent jet, and rarefaction waves. These waves can cause sudden compression or rarefaction effects upon any object encountered in their motion path, and transfer kinetic energy to the object. Therefore, if an experimental animal is placed inside the shock tube, these complex pressure waves will cause more severe and complex injuries that are rarely observed in blast victims, thus leading to false-positive results in the studies of blast TBI mechanism.

## Complex Shock Waves Inside Shock Tube

### Reflected shock waves and mach stem in positive pressure phase

**Figure 1:**
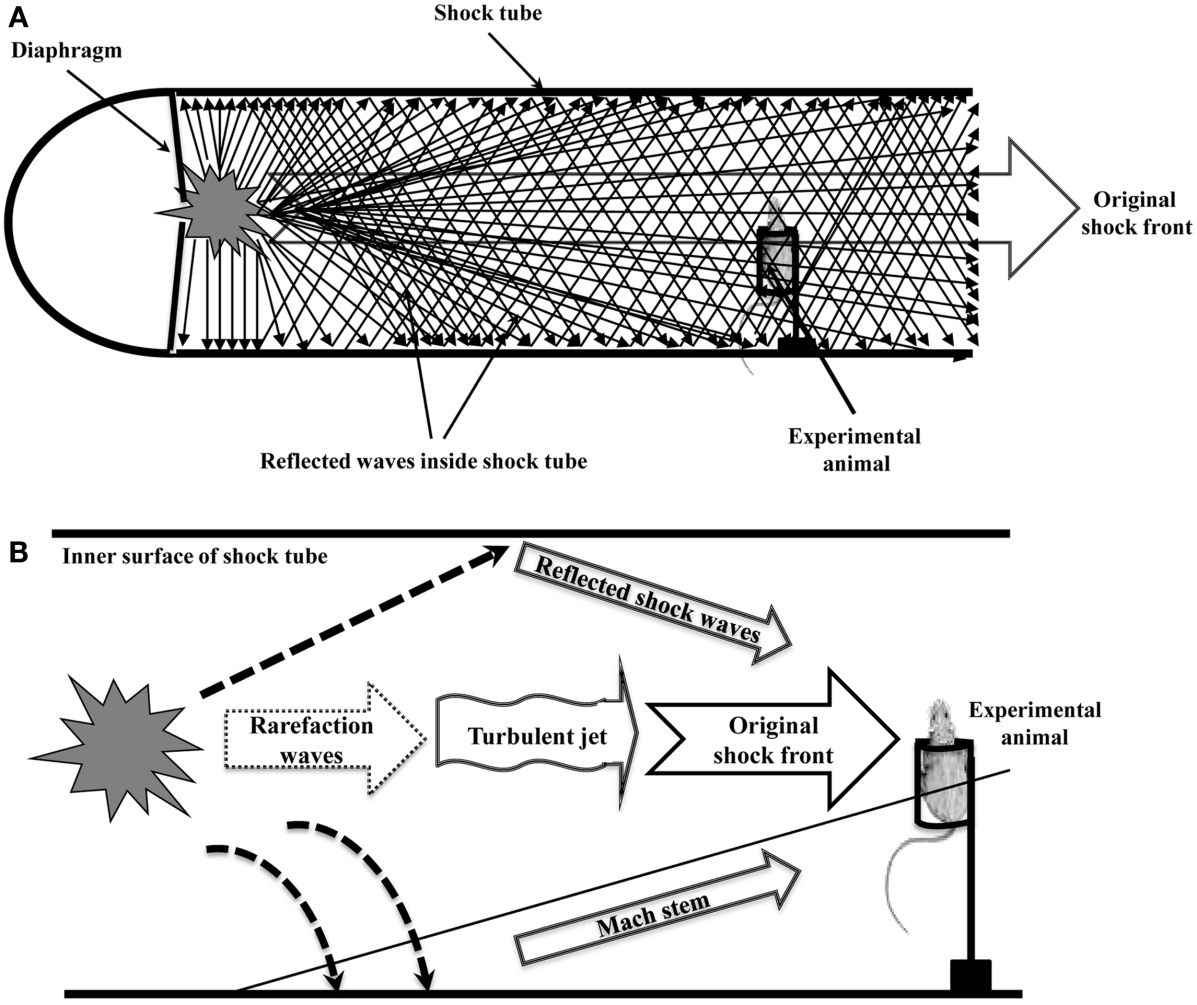
**Complex shock waves inside the shock tube**. **(A)**. A series of reflected shock waves are generated and reinforced when original shock front impinges on the inner surface of the shock tube. **(B)**. Complex shock waves (including the original shock front, reflected shock waves, a Mach stem, an unsteady turbulent jet, and rarefaction waves) transfer kinetic energy to the experimental animal in shock tube, causing severe and complex blast injuries that are rarely observed in the blast victims.

A blast shock wave always propagates as a sphere of compressed gases that rapidly moves outward from the explosive center (1). Because the direction of the shock wave is not parallel to the wall of the shock tube, a series of reflected shock waves are generated and reinforced when the spherical shock wave impinges on the inner surface of the metal tube (Figure 1A). The point of shock impingement will experience the maximum reflected pressure. The pressure of reflected shock wave usually varies with the angle of incidence of the shock wave. In addition to the angle of incidence, the magnitude of the peak reflected pressure is also dependent on the peak incident pressure (2). The increase in the pressure behind the incident shock wave resulting from the growing boundary layer can be magnified by the shock reflection from the closed end of shock tube. The ratio of the pressure behind the reflected shock was up to about 4.5, which suggested stronger reflected waves existed in shock tube (3). Temperature and pressure non-uniformities behind the reflected shock wave were also observed in a shock tube. The reflected wave non-idealities increased in the shock tube due to increased viscous effects, smaller tube diameters, and non-ideal shock formation (4).

When the angle between the inner surface of the shock tube and the incident shock wave is large enough (e.g., more than 20°), the reflected shock waves are not able to turn back the incident shock flow parallel to the inner surface, which then leads to the transition to the Mach reflection (5). Mach reflection consists of three shocks, namely the incident shock, the reflected shock, and a Mach stem. The point where the three shocks meet is called the “triple point” in two dimensions, or a “shock-shock” in three dimensions (6). Mach stem formation occurs when the incident shock waves reflect off of the inner surface of shock tube and the reflection catches up with the original shock front. Different reflected waves can interact with each other to create a high-pressure area that extends from the surface to the “triple point.” Peak pressures in this area can be several times higher than the peak pressure of the original shock front (7). A recent study found that a developed Mach reflection came from a number of reflections off the ceiling and floor of the shock tube before it arrived at the test section. The length of the Mach stem of the reflection pattern is the overall vertical distance traveled by the “triple point.” The fourth wave of the Guderley reflection, which is the expansion wave that follows the reflected shock wave, has been observed (8). The Mach number of the shock wave also increased in the shock tube when the pressure ratio across the diaphragm was increased. After the incident shock reached the end wall of the shock tube, the reflected shock traveled back into the oncoming gases because it is stopped by the end wall of the shock tube (9, 10).

The pressure of reflected shock is always greater than that of the incident shock at the same distance from the explosion. Reflected pressures can be almost 13 times greater than peak pressures of incident shock. In all cases of explosions, the reflected pressure coefficients which equal the ratio of the peak reflected pressure to the peak incident pressure (Cr = Pr/Pi), are significantly greater closer to the explosion (2).

### Unsteady turbulent jet in positive pressure phase

A gas jet in the shock tube is a fast flowing, unsteady turbulent air current. It is caused by the expansion of compressed gases in compressed air-driven shock tube or by rapidly expanding gases in detonation-driven shock tube. The jet of expanding gases behind primary incident shock and reflected waves will apply additional force and transfer momentum to the test object (11). This undesirable “jet effect” will cause additional injuries to the experimental animals in shock tubes and increase the uncertainty and complexity in blast TBI research.

### Rarefaction waves in negative pressure phase

Rarefaction is a decrease in density and pressure in air. It occurs when molecules are given extra space and allowed to expand. A rarefaction wave, also called negative shock wave, is the area of negative pressure following the incident shock wave (12). When an extremely large explosion (such as a nuclear bomb explosion) occurs in the air, the “negative phase” of the shock wave causes a sudden rarefaction of the air surrounding the explosion. This negative pressure region results in a sharp decrease in temperature, thus causing moisture in the air to be condensed in a shell surrounding the explosion (13). Rarefaction wave expands with time and keeps the same overall profile (shape) at all times throughout the wave's movement. The existence of a single-phase vapor rarefaction shock wave is unequivocally demonstrated in the incident flow of the shock tube. The flow is composed of four uniform regions separated by three constant-speed discontinuities: a rarefaction shock, a compression shock, and a contact surface. Entropy jumps and upstream supersonic Mach number conditions were also verified for both rarefaction and compression shock waves (14).

A rarefaction wave may cause much more damages than the incident shock wave due to its cavitation effects (15). A study showed that after an explosive charge was detonated in a semi-confined environment (a semi-confined volume composed of four vertical walls, but without a roof), an incident shock wave propagated in the surroundings. Meanwhile, a rarefaction wave propagated from the contact surface (the surface between the surroundings and the initial gaseous mixture) toward the explosive center. When the rarefaction wave sufficiently decreased the pressure of burnt gas to the ambient pressure, a secondary shock wave was created and traveled toward the explosive center. Once the secondary shock wave reached the explosive center, it was reflected (or imploded) and propagated again in the same direction as the initial incident shock wave. The results suggested that damages caused by rarefaction waves during the negative pressure phase could be more significant than that caused by incident shock waves during the positive pressure phase (16).

## Severe, Rare, and Complex Blast Injuries Induced in the Animals Positioned Inside Shock Tubes

Since 2007, shock tubes have been used to induce blast TBI in rats or mice in 33 different experimental studies. Of the 33 studies, 69.7% (23 studies) reported that the animals were positioned inside the shock tubes, and only 30.3% (10 studies) showed that the animals were placed in contact with the mouth of the shock tubes. Blast injuries that occurred in a cylindrical metal tube (such as sewage, water, oil, or natural gas pipelines) were rarely seen in the blast victims. Most victims were exposed to blasts commonly in a free-field or semi-confined (e.g., the space among buildings or walls) environment. Because of more significant damage effects of reflected shock waves and rarefaction waves inside shock tubes, exposure of animals to shock waves in a shock tube will cause severe, rare, and complex blast injuries. For example, blast shock waves that cause only mild or modest injury in the free field can be lethal if exerted upon animals standing in the shock tube. In addition, a variety of complex physical factors will influence on the observation of experiments if the animals are placed inside a shock tube, which will produce false-positive results and incorrect information about mechanism of blast TBI.

Placing animals inside shock tubes cannot verify if head acceleration or direct cranial transmission of shock waves is the primary injury mechanism of blast TBI, even if the thorax of animal was shielded with a thoracic-protective restraint system (17) or the body (torso) was shielded in a steel wedge (18). The protective restraint system or the body shielding can only shield the wave that travels exactly parallel to the wall of the shock tube, and cannot shield the shock wave that propagates as a shock-compressed gas sphere. If the protective restraint system or the body shielding has only a small opening or crack on the top or the bottom, shock waves (including primary incident shock wave, reflected shock waves, a Mach stem, an unsteady turbulent jet, and rarefaction waves) will easily travel to the animal's torso and cause rapid impact effects on the torso, due to the physical features of spherical wave propagation and reflection in the shock tube (Figure 1B). Therefore, the use of thoracic or body shielding system in the animals placed inside shock tube is unable to rule out the contributions from thoraco-abdominal vascular or hydrodynamic mechanisms to blast-induced TBI.

## Conclusion

Since there are many uncertainties associated with the results obtained from the animals positioned inside shock tubes, the data on injury mechanisms of blast-induced TBI have been difficult to analyze and compare. An adequate experimental design and implementation that can control key parameters of the blast shock waves and mimic critical aspects of blast injury sustained in combat or terrorist explosions are of particular importance for an in-depth and comprehensive study of the biomechanisms, the pathophysiology, and long-term neurological consequences of blast-induced TBI. To reduce exaggerated false-positive results, the experimental animals should been placed outside of the shock tube (at a distance of approximately 10–40 cm from the mouth of the shock tube). An incident shock pressure greater than 20 psi (∼138 kPa) will be necessary to induce blast TBI or other blast injuries in the animals, because an unprotected animal body can survive relatively high incident shock pressure in the free field without experiencing barotraumas. Under the conditions, the protective restraint system or the body shielding may be able to shield the shock waves that will interact with any part (the head, neck, thorax, abdomen, and limbs) of the animal body, thus gaining new insight into the mechanisms of blast TBI. This experimental design will also help evaluate correctly blast protective effects of body armor, helmets, combat boots, and other gears, thus improving personal protection against blast shock wave.
